# Regional versus systemic analgesia in video-assisted thoracoscopic lobectomy: a retrospective analysis

**DOI:** 10.1186/s12871-019-0851-2

**Published:** 2019-10-17

**Authors:** Benedikt Haager, Daniel Schmid, Joerg Eschbach, Bernward Passlick, Torsten Loop

**Affiliations:** 1 Department of Thoracic Surgery, Medical Center, University of Freiburg, Hugstetter Straße 55, 79106 Freiburg, Germany; 2 Department of Anesthesiology and Intensive Care Medicine, Medical Center, University of Freiburg, Hugstetter Straße 55, 79106 Freiburg, Germany

**Keywords:** Minimal-invasive lung surgery, Thoracic paravertebral blockade, Thoracic epidural anesthesia, Patient controlled anesthesia

## Abstract

**Background:**

The optimal perioperative analgesic strategy in video-assisted thoracic surgery (VATS) for anatomic lung resections remains an open issue. Regional analgesic concepts as thoracic paravertebral or epidural analgesia were used as systemic opioid application. We hypothesized that regional anesthesia would provide improved analgesia compared to systemic analgesia with parenteral opioids in VATS lobectomy and would be associated with a lower incidence of pulmonary complications.

**Methods:**

The study was approved by the local ethics committee (AZ 99/15) and registered (germanctr.de; DRKS00007529, 10th June 2015). A retrospective analysis of anesthetic and surgical records between July 2014 und February 2016 in a single university hospital with 103 who underwent VATS lobectomy. Comparison of regional anesthesia (i.e. thoracic paravertebral blockade (group TPVB) or thoracic epidural anesthesia (group TEA)) with a systemic opioid application (i.e. patient controlled analgesia (group PCA)). The primary endpoint was the postoperative pain level measured by Visual Analog Scale (VAS) at rest and during coughing during 120 h. Secondary endpoints were postoperative pulmonary complications (i.e. atelectasis, pneumonia), hemodynamic variables and postoperative nausea and vomiting (PONV).

**Results:**

Mean VAS values in rest or during coughing were measured below 3.5 in all groups showing effective analgesic therapy throughout the observation period. The VAS values at rest were comparable between all groups, VAS level during coughing in patients with PCA was higher but comparable except after 8–16 h postoperatively (PCA vs. TEA; *p* < 0.004). There were no significant differences on secondary endpoints. Intraoperative Sufentanil consumption was significantly higher for patients without regional anesthesia (*p* < 0.0001 vs. TPVB and vs. TEA). The morphine equivalence postoperatively applicated until POD 5 was comparable in all groups (mean ± SD in mg: 32 ± 29 (TPVB), 30 ± 27 (TEA), 36 ± 30 (PCA); *p* = 0.6046).

**Conclusions:**

Analgesia with TEA, TPVB and PCA provided a comparable and effective pain relief after VATS anatomic resection without side effects. Our results indicate that PCA for VATS lobectomy may be a sufficient alternative compared to regional analgesia.

**Trial registration:**

The study was registered (germanctr.de; DRKS00007529; 10th June, 2015).

## Background

Video-assisted thoracic surgery (VATS) is considered as the standard minimal invasive surgical procedure for anatomic lung resections [1]. The advantages of VATS compared with open thoracotomy include faster recovery, reduced perioperative pain intensity, and decreased postoperative morbidity [2–4]. Nevertheless, persistent pain after VATS affects the ability to cough, impairs deep breathing and lung function, resulting in cardiorespiratory complications (> 15%), delayed recovery and increased costs [2].

The optimal perioperative analgesic strategy after VATS lobectomy remains contradictory. Thoracic epidural analgesia (TEA) is commonly considered as the gold standard for pain relief after open thoracotomy and is preferred by the majority of clinicians. In times of enhanced recovery and fast track concepts after surgery thoracic paravertebral block (TPVB) is an upcoming regional anesthesia technique in thoracic anesthesia. It can be used as single injection or continuous technique. However, there are also anesthesiologists in the field of thoracic anesthesia preferring patient-controlled analgesia (PCA) instead of the regional anesthesia techniques [5–7]. Regional analgesia techniques such as TEA may be not suitable for all patients for technical reasons or anticoagulative drug therapy and may be associated with numerous risks (e.g. dural perforation, spinal cord damage by formation of hematoma, infection and abscess; hypotension; urinary retention) [8, 9]. The role of TPVB in this context has not been as clear but shown to be effective for pain relief with less hemodynamic side effects than TEA [5, 10–15]. As there is no evidence for one superior regional technique for pain relief after VATS, single-shot or continuous TPVB may be a suitable alternative to TEA or systemic opioid application. Intravenous patient-controlled analgesia with morphine analogue (PCA) is a widely used, simple, and convenient method [16].

After implementation of VATS as a standard for anatomic lung resection in our department the procedure-specific pain protocol was based on multimodal systemic analgesia with non-opioid and opioid drugs. Although less invasive, the thoracoscopic approach, resulted in unexpectedly high intensity of postoperative pain [17]. In this respect, regional analgesia (i.e. TEA or TPVB) was considered to be the crucial component of multimodal postoperative pain management in our department.

Accordingly, we wanted to analyze and undertook a retrospective analysis to establish whether thoracic regional analgesia (TPVB with ropivacaine alone or TEA) would provide improved analgesia compared with systemic analgesia with parenteral opioids and non-steroidal analgesics leading to a reduction postoperative pulmonary complications such as atelectasis, pneumonia, hypoxia or pulmonary dysfunctions.

## Methods

The study was approved by the local ethics committee (AZ 99/15) and registered (germanctr.de; DRKS00007529). Inclusion criteria were age older than 18 years and anatomic lung resection via VATS approach. Exclusion criteria were conversion to open thoracotomy, non-anatomic (“wedge”) lung resections and additional chest wall resections. Data were retrospectively collected between July 2014 und February 2016. Patients signed a written informed consent approving their data could be used for scientific purposes. From July 2014 until January 2015 all patients received either a TPVB or a TEA. From January 2015 the perioperative analgesia was changed by interdisciplinary institutional decision and included systemic opioid application with piritramide as patient controlled analgesia (PCA; Graseby 3300; PCA Syringe Pump; SMITHS MEDICAL INTERNATIONAL LIMITED, Watford, Hertfordshire, United Kingdom).

### Anesthetic management

The perioperative anesthetic regimen is standardized. Pre-medication before arrival in the operating room was performed with midazolam (3.75–7.5 mg p.o.). The responsible consultant preoperatively decided indication and choice for TPVB or TEA. Three experienced anesthesiologists performed all TEA and TPVB following the same protocol. Patients in the group TEA received an epidural catheter, using an 18G Tuohy needle, the epidural catheter (20G) will be placed at T4/5, T5/6, or T6/7 interspace (depending on the site of surgery) using the midline approach and hanging drop technique [18]. Epidural block analgesia was induced with 10 ml of ropivacaine 0.2% and sufentanil (0.2–0.3 μg/kg, maximally 25 μg) administered as three separate injections, followed by a continuous infusion of ropivacaine 0.2% and sufentanil 0.5 μg/ml with a fixed infusion rate at 8 ml/h until 24 h after operation. The paravertebral space is located by using the technique described as previously described [13, 19]. After introduction of the catheter (3 cm into the paravertebral space), gentle aspiration, and test dose application (3 ml of ropivacaine 0.5% with adrenalin (5 μg/ml)), thoracic paravertebral blockade (TPVB) was induced with 30 ml ropivacaine 0.5% with adrenaline (5 μg/ml) followed by continuous paravertebral application of ropivacaine 0.2% (fixed infusion rate 8 ml/h). The patients with systemic analgesia received an intravenous PCA, which was started immediately postoperative with a PCA device programmed to deliver piritramide i.v. (bolus dose of 1.5 mg, with a lockout time of 5 min and restricted total dose of 40 mg/4 h). After an initial dose of 0.4–0.6 μg/kg of sufentanil, additional bolus doses of 0.1–0.2 μg/kg of sufentanil are administered as needed. Induction of anesthesia was performed by a target-controlled infusion (TCI) of with propofol (Propofol 1% MCT & Injectomat® TIVA Agilia, Fresenius-Kabi GmbH, Bad Homburg, Germany) at plasma concentrations of 2–4 μg*ml^− 1^. The Bispectral Index of the encephalogram (BIS) was monitored (BIS® A-2000 monitor, Aspect Medical Systems, Newton, MA, USA) and the propofol concentration was decreased to 2.2 μg/ml at the lowest, if the BIS value decreases to below 30. Propofol concentration was increased to 4 μg*ml^− 1^ to avoid arterial blood pressure values above 20% of baseline and BIS values above 60. Core temperature was kept above 36.0 °C using a forced-air warming system. All patients were endotracheally intubated with a double-lumen endobronchial tube for one-lung ventilation.

### Surgical management

VATS lobectomy was performed using a utility incision of 5 cm length entering the 4th intercostal space regardless which lobe was resected. Two further incisions of 1–2 cm were placed in posterior and anterior axillary line at level of the diaphragm in the 7th or 8th intercostal space. At the end of the procedure a 24 Fr chest tube was placed exiting the anterior lower incision in the 7th or 8th intercostal space. Following the same protocol two thoracic surgeons performed all procedures (B.P., B.H.). Chest tubes were removed when there was no air leakage for 6 h and the pleural fluid amount for 24 h did not exceed 200 ml.

### Postoperative pain management

In the author’s institution surgical patients were preoperatively instructed in the use of the visual analog scale (VAS). The VAS Score consisted of an unmarked 10 cm line, with 0 cm representing no pain and 10 cm the worst imaginable pain. Postoperatively all patients received a basic analgesic therapy containing either metamizole 4 × 1 g per day or acetaminophen 3 × 1 g depending on comorbidities (i.e. renal/liver dysfunction, allergies) and oxycodone 2 × 20 mg per day directly in the intermediate care unit. When the pain intensity exceeded 3 cm a bolus of piritramide 1.5 mg was applicated and repeated until the pain level decreased below 3 (VAS) again. Patients of the PCA group were directly connected to an i.v. PCA device, delivering piritramide bolus doses of 1.5 mg with a lockout time of 5 min and a total dose of 40 mg in 4 h. Nursing staff assessed pain intensity on the intermediate care unit in intervals of 4 h until 24 h after surgery, following once a day until 5th postoperative day. Morphine equianalgesic conversion was calculated using the calculator based on the American Pain Society guideline (http://americanpainsociety.org/uploads/education/PAMI_Pain_Mangement_and_Dosing_Guide_02282017.pdf).

### Postoperative non-pain management

Heart rate and arterial blood pressure were monitored continuously for the first 24 h postoperatively on the intermediate care unit and every 8 h after discharge to the ward. Hypotension was defined by mean arterial pressure below 60 mmHg. A chest x-ray was routinely performed immediately after the operation and on the day following the removal of the chest tube. Radiologic infiltrates were defined by the written result from a consultant of the department of radiology. Pneumonia was defined by either radiologic proven infiltrate with necessity of antibiotic treatment or microbiological proof of bacteria making an antibiotic treatment necessary. A blood cell count was routinely performed on POD 1, leukocytosis was observed when leucocyte count exceeded 9.800/ml. Pruritus, postoperative nausea and vomiting (PONV) and paresthesia were checked twice daily during the morning and afternoon round of the medical staff on the intensive care unit.

### Outcome measures

Primary endpoint was the postoperative pain intensity assessed by the VAS in cm at different times after the procedure. VAS scores (0–10) were assessed by the nursing staff at the beginning of the shift routinely at rest and during coughing on ICU. When transferred to the ward after 24 h, pain scores were documented once daily during the morning round of the nursing staff. Secondary outcome parameters were pulmonary (i.e. atelectasis, pneumonia, pulmonary embolism and respiratory failure) and surgical complications (i.e. leukocytosis, time to chest tube removal, pleural effusion) as well as side effects of the analgesic therapy (hypotension, pruritus, paresthesia, PONV). Atelectasis was defined by radiological criteria, pneumonia as fever, radiologic infiltration, positive microbiology or leukocytosis requiring antibiotic treatment.

### Statistical analyses

Data were presented as mean and standard deviation (± SD) or median and IQR if not indicated otherwise. Patient characteristic data were compared by analysis of variance (ANOVA) for multiple comparisons with Tukey post-hoc test. Comparisons of serial measurements (VAS for pain) were performed with repeated-measures ANOVA. Ranked data were analyzed with the Kruskal–Wallis and Mann–Whitney U-tests when appropriate. Categorical data were examined by Fisher’s exact or Chi-square test. Probability values under 0.05 were considered significant.

## Results

From July 2014 to February 2016, 103 patients who underwent VATS lobectomy for oncologic reasons were examined retrospectively and initially included. In 62 patients analgesia was performed by regional anesthesia (28 patients with TPVB and 34 with TEA). From May 2015 patients were scheduled without regional anesthesia due to change in local procedures and 41 patients underwent VATS lobectomy with systemic opioid-based analgesia (PCA). Four patients were excluded due to conversion to systemic analgesia because of postoperative catheter dislocation, inadequate data sheet or open thoracotomy. The patient’s demographic data are described in Table 1.

**Table 1:** Patient and surgical characteristics

	TPVB (*n* = 25)	TEA (*n* = 31)	PCA (*n* = 41)	Total (*n* = 97)	*P* value
*Sex*					
female	7	9	22*	38	*P* < 0.04
male	18	22	19	59	
*Age* (median/range) (yr)	69 (45–81)	72 (46–88)	68 (43–81)	70 (43–88)	0.865
*ASA Score* (n)					0.520
I	0	0	0	0	
II	4	1	9	14	
III	20	29	31	80	
IV	1	1	1	3	
*Type of surgery*					0.225
Lobectomy	20	24	29	73	
Segment resection	3	6	12	21	
Pneumonectomy	2	1	0	3	

*TPVB* Thoracic paravertebral blockade, *TEA* Thoracic epidural analgesia, *PCA* Patient controlled analgesia, *ASA* American Society of Anesthesiology;

*p-value < 0.05

**Fig. 1:**
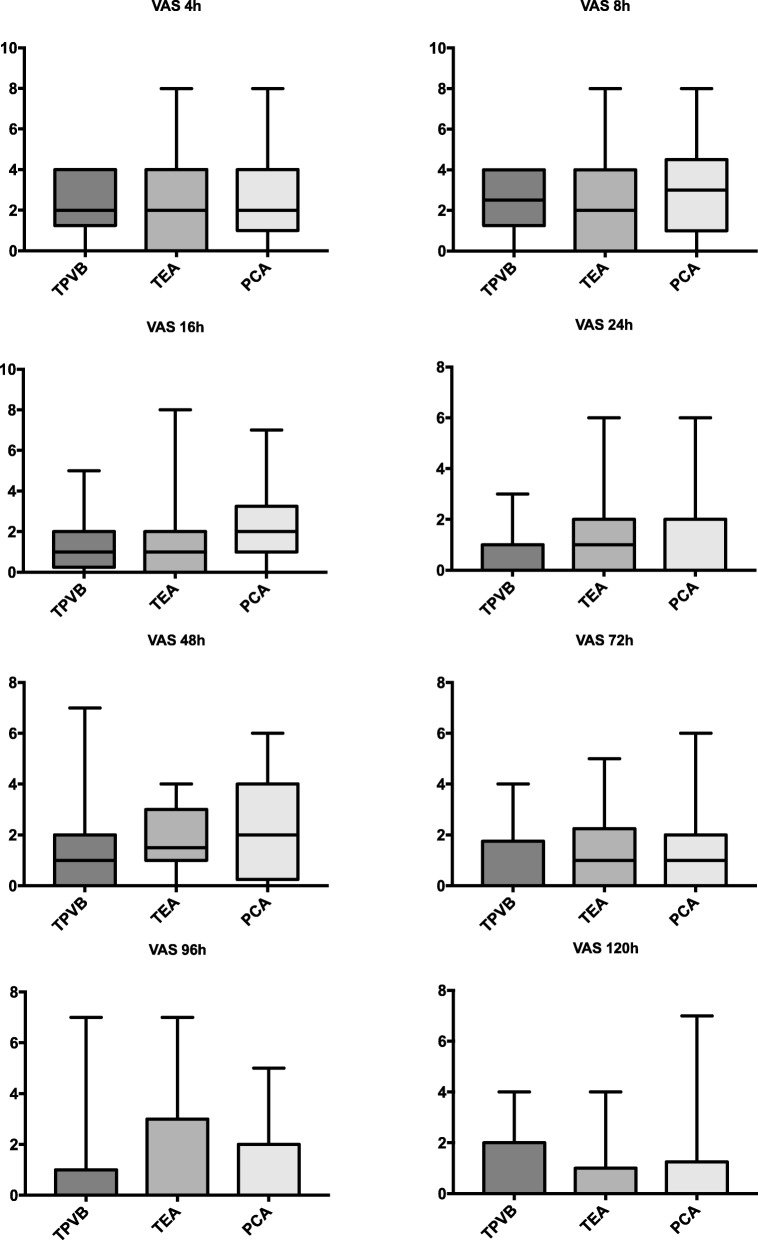
VAS at rest. TEA. thoracic epidural analgesia; TPVB, thoracic paravertebral blockade; PCA, patient controlled analgesia. Boxplots show median, 25/75th, and 5/95th percentiles

**Fig. 2:**
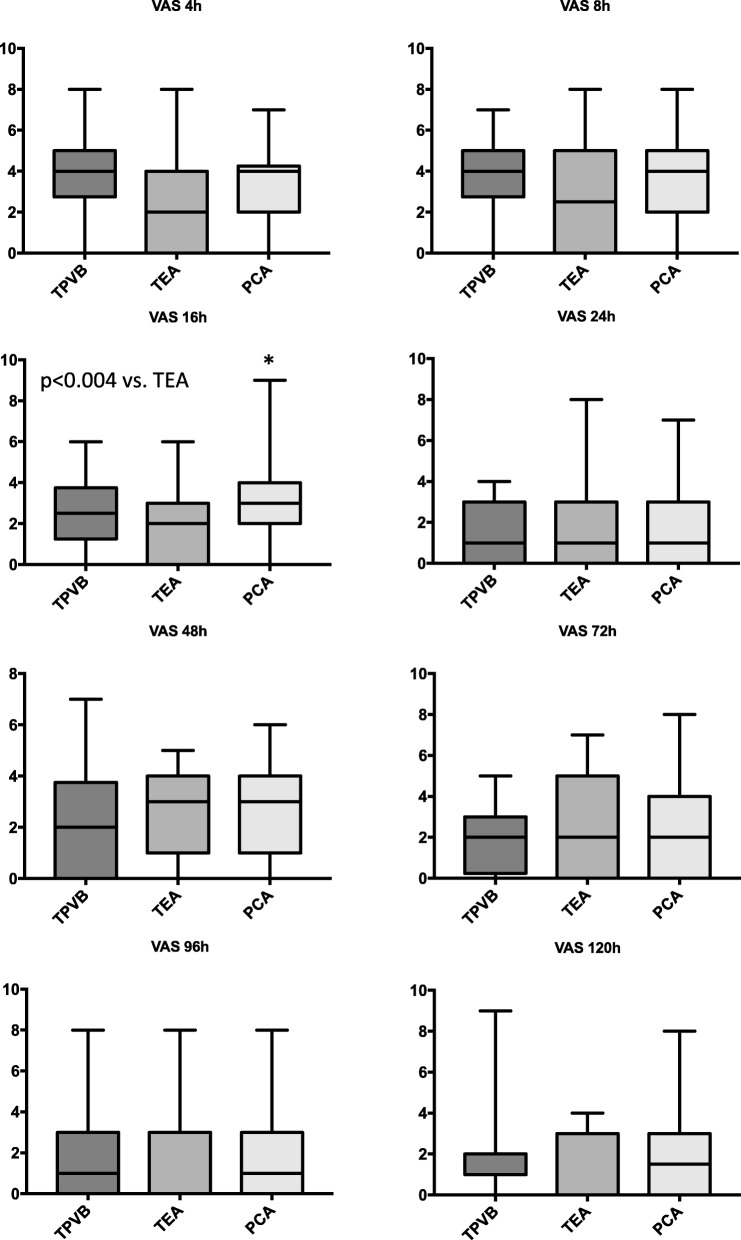
VAS during coughing. TEA, thoracic epidural analgesia; TPVB, thoracic paravertebral blockade; PCA, patient controlled analgesia. Boxplots show median, 25/75th, and 5/95th percentiles. (*p < 0.004 vs. TEA)

**Fig. 3:**
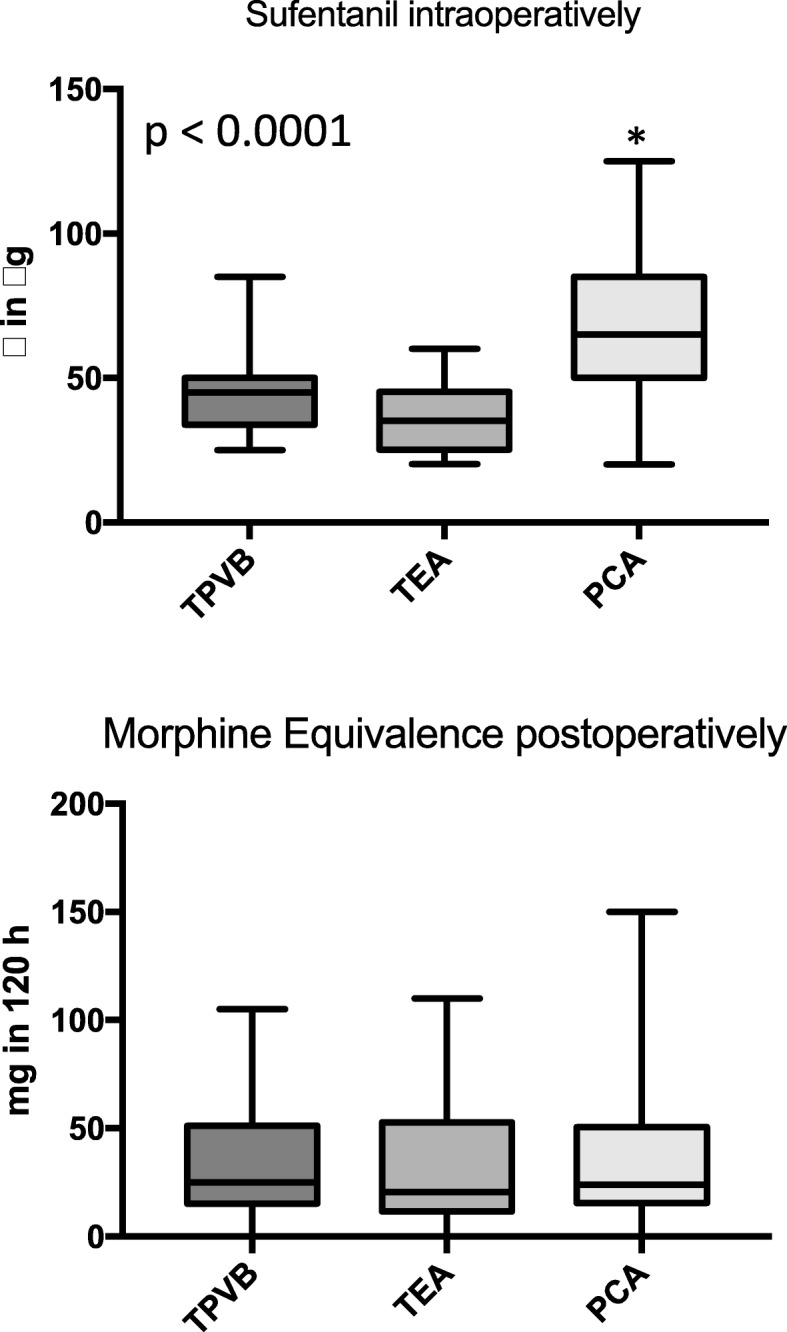
Opioid consumption. TEA, thoracic epidural analgesia; TPVB, thoracic paravertebral blockade; PCA, patient controlled analgesia. Boxplots show median, 25/75th, and 5/95th percentiles. (*p < 0.0001 vs. TEA and TPVB)

Mean VAS score was measured below 3.5 in all groups showing effective perioperative analgesia (Figs. 1 and 2). The VAS values at rest were comparable between all groups, VAS values during coughing were also effective and comparable with except higher in patients with PCA compared to TEA after 16 h postoperatively (Fig. 2). The intraoperative dose of sufentanil was significantly higher in the PCA group (Fig. 3; *p* < 0.0001; mean dose 67 ± 4 μg for the PCA group vs. 47 ± 3 μg vs. patients with TPVB and 34 ± 2 vs. patients with TEA). The postoperative morphine equivalence dose applicated postoperatively until postoperative day 5 was comparable in all groups (Fig. 3: median (25–75%) in mg: 25 (15–51) (TPVB), 20 (11–52) (TEA), 24 (16–51) (PCA); *p* = 0.60).

Secondary endpoints showed no difference between the three groups (Table 2). Hemodynamic complications like hypotension demanding a vasopressor therapy were observed in 4 patients each with TPVB and PCA compared to 7 patients with TEA (Table 2). There was no pulmonary complication such as pulmonary embolism or respiratory failure. Pneumonia was diagnosed in 3 patients with TPVB, two patients with TEA, and 6 patients in the PCA group (Table 2). Pruritus was observed in one patient with TPVB and in 3 with TEA. No patient with PCA had pruritus (Table 2).

**Table 2:** Secondary Endpoints

	TPVB (*n* = 25)	TEA (*n* = 31)	PCA (*n* = 41)	Total (*n* = 97)	*P* value
*Hemodynamics (n)*					
Hypotension	4	7	4	15	0.445
Hypertension	23	28	35	86	0.976
Pneumonia (n)	3	2	6	11	0.617
Pruritus (n)	1	3	0	4	0.149
Leucocytosis (n)	16	13	21	50	0.653

*TPVB* Thoracic paravertebral blockade, *TEA* Thoracic epidural analgesia, *PCA* Patient controlled analgesia

## Discussion

This retrospective study analyzed two regional analgesic concepts (TPVB and TEA) and one systemic concept via opioid application using a PCA for postoperative analgesia in patients undergoing VATS lobectomy or VATS anatomic resections. The main findings can be summarized as follows: (i) TPVB, TEA and systemic analgesia provided effective analgesia (VAS < 4) during the perioperative period; (ii) the postoperative pain relief was comparable with similar opioid doses in all groups with the exception of 16 h postoperative while coughing favoring regional anesthesia; (iii) secondary outcome measures as respiratory or surgical complications did not differ among the three groups.

There are two major issues influencing the postoperative fast track concept in thoracic surgery demanding an optimal pain relief. First, early effective pain relief augments early mobilization with possible reduction of pulmonary complications (e.g. atelectasis, pneumonia) leading to early discharge and reduced health costs [20]. Secondly, thoracic surgery (especially thoracotomy) is associated with one of the highest incidences of chronic pain syndrome (up to 50%) [21]. There was a significantly lower incidence for VATS procedures, though still a number of patients (34%) emerge from VATS thoracic surgery suffering from chronic pain [22]. Placement of trocars and utility incision can cause intercostal nerve injury and pleural irritation as thoracotomy does, promoting pain transmission to the central nervous system leading to pain memory. Effective block of neural afferents can reduce acute postoperative pain and avoid the development of a pain consciousness [18].

For VATS resections the reports on different analgesic strategies are still very heterogeneous [7, 23, 24]. TEA is established as gold standard for thoracotomy and is widely used for anatomic VATS resections as well as intravenous delivery of opioids via PCA [25–27]. The results of meta-analyses and reviews demonstrated that TPVB with local anesthetics has a comparable efficacy and a higher safety profile [14, 28, 29]. Kosinski et al. compared continuous epidural with paravertebral analgesia in a prospective randomized study and demonstrated a favorable effect for the paravertebral block on pain scores on the POD 1 and 2. The consumption of opioids was comparable and the authors found a higher rate of side effects for example urinary retention and hypotension in the TEA group. Beyond that the use of paravertebral block was recommended due to the better safety profile and comparable analgesic effect [30].

In this study, TEA, TPVB, and PCA provided a comparable pain relief. Two studies compared TEA with systemic opioid analgesia for thoracoscopic lobectomy. Kim et al. demonstrated in a non-blinded RCT of 37 patients that there were no differences in pain scores, supplementary analgesic requirements or adverse events [25]. Yie et al. investigated 105 patients retrospectively and found a lower VAS in the TEA group, but only on POD 2. Incidence of dizziness was shown to be higher in the morphine group on POD 1, whereas pruritus was higher in the TEA group on POD 2 and 3 [26].

There were no differences with respect to the pain intensity between opioid PCA and TEA or TPVB. The cost implications of a regional analgesia concept, TEA or TPVB, compared with i.v. analgesia is well documented. The cost difference is mainly caused by the professional manpower costs and the treatment of complications [31].

In addition to the comparable pain relief we found no difference between the secondary outcomes (e.g. pulmonary complications, surgical complications, PONV) not supporting our hypothesis that there would be less pulmonary complications using a regional anesthetic procedure.

This study had some limitations. The trial was not a prospective, randomized, and double-blinded clinical study, but technique and expertise in TEA or TPVB was very homogeneous and performed exclusively by three consultants and reflects clinical practice. In addition, pain assessment was routinely evaluated only at the described time points. Secondly, the small sample size limited the possibility of drawing a definitive conclusion. Thirdly, the sample size of the study was not calculated due to the retrospective design and therefore to small to evaluate the secondary outcomes of respiratory function, pulmonary complications, nausea and vomiting, degree of sedation, hypotension, and pruritus. Finally, assessment of the level of analgesic effect or sensory block of the TEA and TPVB were not routinely performed.

## Conclusions

In conclusion, our findings indicated that application of systemic opioids via PCA device was an effective and acceptable alternative to regional anesthesia with TEA or TPVB for postoperative pain relief for patients undergoing VATS lobectomy.

## Data Availability

The datasets generated and analyzed during the current study are not publicly available due institutional restrictions but are available from the corresponding author on reasonable request.

## Abbreviations

ANOVA: Analysis of variance
PCA: Patient-controlled analgesia
POD: Postoperative day
PONV: Postoperative nausea and vomiting
TCI: Target-controlled infusion
TEA: Thoracic epidural analgesia
TPVB: Thoracic paravertebral block
VAS: Visual analog scale
VATS: Video-assisted thoracic surgery
